# Comparison of Imaging Models for Spectral Unmixing in Oil Painting †

**DOI:** 10.3390/s21072471

**Published:** 2021-04-02

**Authors:** Federico Grillini, Jean-Baptiste Thomas, Sony George

**Affiliations:** The Norwegian Colour and Visual Computing Laboratory, Department of Computer Science, Norwegian University of Science and Technology, 2815 Gjøvik, Norway; jean.b.thomas@ntnu.no (J.-B.T.); sony.george@ntnu.no (S.G.)

**Keywords:** spectral imaging, imaging models, spectral unmixing, pigment mapping

## Abstract

The radiation captured in spectral imaging depends on both the complex light–matter interaction and the integration of the radiant light by the imaging system. In order to obtain material-specific information, it is important to define and invert an imaging process that takes into account both aspects. In this article, we investigate the use of several mixing models and evaluate their performances in the study of oil paintings. We propose an evaluation protocol, based on different features, i.e., spectral reconstruction, pigment mapping, and concentration estimation, which allows investigating the different properties of those mixing models in the context of spectral imaging. We conduct our experiment on oil-painted mockup samples of mixtures and show that models based on subtractive mixing perform the best for those materials.

## 1. Introduction

In the past decades, many research bodies have specialised in the field of spectral imaging, an acquisition technique that allows the pixel-wise evaluation of the radiance spectrum of a scene. Depending on the number of spectral channels or the spectrum interval spanned, the technique is referred to as Multispectral (MSI) or Hyperspectral Imaging (HSI).

Remote sensing has been one of the first research areas that exploited HSI, with applications aimed in the fields of agriculture [1], military [2], and mineralogy [3]. Spectral Unmixing (SU) is one of the most studied applications within remote sensing [4], since it allows the identification and mapping of specific materials, denominated endmembers, by decomposing a spectrum into fundamentals, according to a pre-determined imaging model.

To have material-specific endmembers, the effect of the illumination is discarded by calibration, and the wavelength-dependent spectra of reflectance factors ρ(λ) are treated. Each endmember is assumed to be present in a specific mixture with a relative concentration α. The concentrations related to the *q* possible endmembers of a scene are grouped in the concentration vector $C={\left(\alpha_{1},\alpha_{2},\ldots ,\alpha_{q}\right)}^{T}$, which is subject to two physical constraints: the non-negativity constraint (NC) $\alpha_{i}\geq 0,\forall i\in \left\{1,\ldots ,q\right\}$, and the sum-to-one constraint (SC) $\sum_{i=1}^{q}\alpha_{i}=1$. In several applications, to grant a certain degree of flexibility to the algorithms, the two constraints (particularly SC) can be relaxed to allow a margin of tolerance. SU eventually boils down to an optimisation problem, where the spectral library E (provided or extracted from the scene) is used to decompose a target spectrum *Y* into *q* components and their relative element of the concentration vector *C*:

(1) $$Y_{\left[N\times 1\right]}=f\left(E_{\left[N\times q\right]},C_{\left[q\times 1\right]}\right)$$

in which *N* represents the number of spectral bands.

In remote sensing, the linear model (Equation (2)) has been extensively used to perform SU [5,6,7].

(2) $$Y\left(\lambda \right)=\sum_{i=1}^{q}\;\rho_{i}\left(\lambda \right)\;\alpha_{i}$$

The rationale behind this choice resides in the fact that satellite images are limited in optical resolution and the surface on earth represented by a pixel can be a few meters to several dozen meters. Two materials that are physically separated on the ground-level may end up being represented in the same pixel, at the camera level. In this instance, the linear model is a fair approximation since the amount of light incoming from a material is proportional to the sensor surface that the material itself covers. The state of the art on SU comprehends linear and non-linear approaches, as well as supervised and unsupervised methods. For an exhaustive review on the topic of SU, refer to [4].

Over the recent years, several research lines such as the food industry [8,9,10], medical imaging [11], biology [12], and cultural heritage [13,14,15,16,17], have started to exploit spectral imaging for close-range applications. Contrarily to remote sensing, when spectral imaging is applied to targets found in close-range with respect to the camera sensor, it is safe to assume that the optical resolution of the system is powerful enough to discern physically separated objects. Therefore, in this context, the usage of the linear model to perform spectral unmixing might be a too coarse approximation, and non-linear models should be preferred.

In the context of Cultural Heritage (CH), the optical mixing problem can be intuitively extended to the mixing of pigments in artworks. HSI is a well-appreciated technique in the field of conservation science, since it enables the study of CH artefacts in a way that is non-invasive and non-destructive, features that are imposed by the ethical guidelines issued by the CH community. HSI can be adopted to perform monitoring of artefacts [18], pigment mapping [19], forgery detection [20], and rejuvenation of paintings [21].

Pigment mapping (PM) in its standard form allows the spatial identification of pigments across the surface of a painting, resulting in binary maps. Moving a step forward, with the application of SU, it is possible to retrieve abundance maps, as gray-level images for example, considering the spectral signatures of the pure pigments as endmembers in the unmixing problem. Examples of recent works on PM have seen the inversion of the linear model, to map the pigments of Edvard Munch’s *The Scream* [19,22], and to perform pigment identification [23] using the sparse SUnSAL approach [24]. Recently, PM has been tackled with Deep Learning approaches as well [25,26,27]. Those mentioned works, however, focus on improving the performances of the application, rather than on the imaging model that generated the data. Thus, the aim of this study is not to compare against the literature in terms of pigment detection accuracy, but rather the investigation of the role of the imaging models.

In the present study, mockup samples of mixtures in various concentration ratios were realised using seven commercially available pigments that mimic a possible palette of the Renaissance period, and then acquired with an HSI setup. Seven mixing models are selected and adapted from the literature to assess their properties through an evaluation protocol composed of three steps. The first stage involves the forward-feeding of the models with all the information contained in the ground truth of the mockup samples, to evaluate the spectral accuracy of the outputs. In the two following steps, the models are inverted in two SU tasks that differ in the amount of prior information provided regarding the ground truth, with the aim of evaluating the ability of each model in identifying and estimating correctly the pigments and their relative concentrations.

The models are selected based on criteria that aimed to describe specific underlying imaging configurations, with the goal of comparing them through the proposed evaluation protocol. In the particular instance of the investigated imaging models, the results show that models having a subtractive nature are more accurate under several standpoints: spectral reconstruction, accuracy in pigment identification, and precision in determining the relative abundances. A validation test of pigment mapping is run on a mockup painting realised with the same set of pigments, confirming the observation made on the dataset of mixture samples.

We have investigated the models on a preliminary mockups set [28], and applied the best model to the mockup painting of this work [29]. The present article strengthens and refines our preliminary observations.

The remainder of this paper is organised as follows: Section 2 introduces the investigated imaging models and provides detailed descriptions of the experimental setup and the methodologies adopted, while Section 3 shows the results, and Section 4 includes a few concluding remarks and future work.

## 2. Materials and Methods

### 2.1. Imaging Models

**Figure 1:**
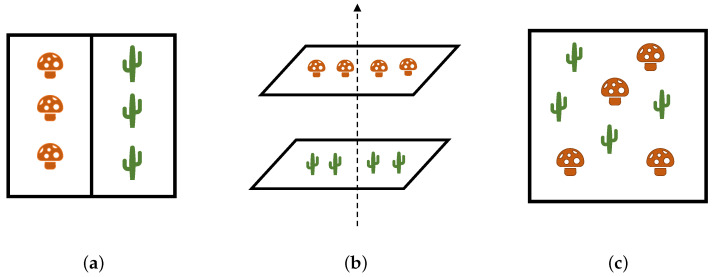
Three possible configurations of pigment mixing. (**a**): Optical blending occurs when two materials are physically separated but mixed at the camera level. (**b**): A layered structure assumes that light is transmitted and reflected according to the properties of the different layers. (**c**): In the intimate mixing the components are not physically discernible.

Equation (1) shows that an imaging model combines the reflectance factors of the endmembers, with their relative concentrations. In this perspective, we consider three possible cases on how the pigments (or endmembers in general) might be mixed when represented as an individual pixel. Figure 1 schematically reports such configurations, which can be smoothly applied to the case of pigment mixing and lead us to propose the use of specific mixing models.

Optical blending (Figure 1a) occurs when the paints are physically separated on the canvas, but due to the lack of spatial resolution by the acquisition system, they are eventually represented with a single pixel value. An artistic example can be found in Pointillism, in which many dots of different paints are applied on the canvas to produce the perception of a uniform colour [30].

In a speculative layered structure, pigments are applied one over the other (Figure 1b). Having multiple pictorial layers is not an unusual instance: often artists covered their *pentimenti* with more details, but also adopted the *fat over lean* technique, applying first the layers with lower oil content, and the fatter layers once the previous ones have dried out [31]. Technically, pigments float in the binding material (linseed oil, egg tempera, acrylic, etc.) creating a suspension [32] in which the different powder particles are scattered across the volume. Therefore, the pigment powder should be separable from the binder. However, the considered scale is observable only with advanced microscopic instrumentation, and for this reason, the mixture of pigments can be considered *intimate* [33] (Figure 1c), borrowing the terminology from the remote sensing community. In the technique called *alla prima* (literally, *on the first attempt*), different pigment powders are blended and applied onto the canvas [32]. In the next paragraphs, these mixing configurations are associated with imaging models, explaining the rationale behind each choice.

The selected imaging models are labelled with the notation $M_{x}$, and their equations are reported at the end of the section in Table 1 grouped into 3 categories: additive (A), subtractive (S), and hybrid (H). To ease the reading, the notation related to the wavelength-dependency has been dropped. All models are assumed to strictly comply with the non-negativity and sum-to-one constraints. All models assume diffuse reflections and do not handle specularities nor Bidirectional Reflectance Distribution Functions (BRDF) [34].

The linear model (Equation (2)) is considered and labelled as $M_{1}$. To oppose it, the subtractive model based on the weighted geometric mean [35] is considered and labelled with the notation $M_{2}$. Given its non-linearity, this model is selected to represent the instance of intimate mixing displayed in Figure 1c. Three hybrid models defined between $M_{1}$ and $M_{2}$ are selected in this work: the Yule–Nielsen model ($M_{3}$) [36], which was originally proposed to study halftoned colours in printing, the Additive-Subtractive model ($M_{4}$) [37], and the Subtractive-Additive model ($M_{5}$) [37]. All three hybrid models are modulated by the mixing constant $\tau \in \left[0,1\right]$, which determines the weight of one model or the other on the output, as the models approach $M_{1}$ and $M_{2}$, when τ is set to 1 and 0 (asymptotically for $M_{3}$), respectively. The underlying physical model is well described in [37], and it is represented as a combination of layers and adjacent areas containing different endmembers.

The layered configuration displayed in Figure 1b has been historically modelled by the Kubelka–Munk theory [38,39]. However, there exists a framework in digital image processing that aims to achieve meaningful image reproduction considering images as transmission filters. The Logarithmic Image Processing (LIP) framework [40] transforms the standard operators of addition, subtraction, multiplication, and power to better mimic the human visual system, following the Weber–Fechner law of brightness perception [41]. LIP can be exploited to draw parallelism to the layered structure of pictorial layers applied onto the canvas. Furthermore, another parallelism can be made by observing the fact that both reflectance spectra and images in LIP must comply with an upper bound limit at a value μ, which is for example 255 for 8-bit images, and 1 for reflectance factors (if fluorescence effects are neglected). By utilising the set of rules provided by LIP, the linear and subtractive models $M_{1}$ and $M_{2}$, are transformed into the models labelled as $M_{6}$ and $M_{7}$, respectively. It is worth mentioning that the LIP addition operation features the commutative property, so the order of the layers does not matter. The same cannot be said for consecutive pictorial layers, as it is possible to assume that the outer layers play a more significant role in the perception of the resulting colour. Nevertheless, the LIP framework is considered an appropriate approximation of the layered configuration.

We decided to not include in our study models that explicitly solve the radiative transfer equation, although some might be very relevant for future work, for example, the Kubelka–Munk or the four flux [42] models. One very practical reason for that is the need of measuring the scattering and absorption coefficient of the endmembers. This would be feasible [43], but fairly cumbersome, and we had not the possibility to do that in our study. Other reasons to not use those models are related to the several conditions of applicability [44] and the assumptions that need to be verified (semi-infinite or infinite material, distribution of pigments in the binding material, number of layers, etc.). Although some of those hypotheses are certainly assumed implicitly in the models we studied, we do not need to verify them according to the fact that the models are directly embedded into the imaging process.

### 2.2. Mockup Samples Realisation and Imaging Setup

Mockup samples of mixtures were realised using seven pigments manufactured by Kremer [45], composing a palette relevant to oil painting in the Renaissance period (Table 2). The pigment Kremer White was chosen to replace Lead White, which was commonly used in the past but is not sold nowadays due to its high toxicity.

**Figure 2:**
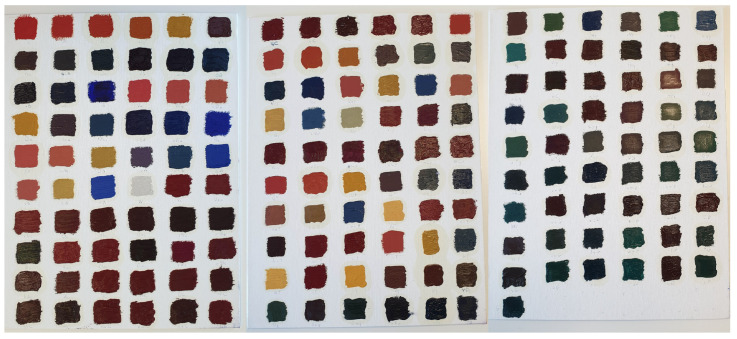
Set of mockup samples realised for the experiment. The 175 painted patches are ordered according to a script, not considering perceptual similarities. Reproduced from [29] with permission from the International Colour Association (AIC).

Pre-primed stretched canvases made of linen of size 35×27 cm are used as supports. Although the canvases are sold already primed, an ulterior layer of gesso in acrylic base is applied to facilitate the adhesion of the pictorial layer. To compose the mockups, only the mixtures including up to 3 pigments are considered, since in traditional oil painting this is usually the ceiling number of pigments per mixture [46]. Mixtures including two pigments are performed for all combinations of the seven endmembers in concentration ratios of 1:1 and 2:1. When mixtures of three pigments are considered, all the combinations of ratios 2:1:1 are realised. A total of 175 mixtures has been performed (Figure 2).

To obtain a faithful ground truth of concentrations, the pigments in powder form are weighed on a precision scale with 0.005 g sensitivity. This means that the concentrations refer to relative proportions of masses and not volumes, indeed the pigments are bound to linseed oil at a later stage. The mockup samples are named after the pigments that compose the mixture, considering the information regarding the relative concentration ratios as well. Using the labels contained in Table 2, a mixture can be named in the following ways:

- X, endmember, 100% of that pigment,
- XY, ratio 1:1 between the 2 pigments,
- Xy, ratio 2:1, with X being the most concentrated,
- Xyz, ratio 2:1:1, with X being the most concentrated.

Each sample patch has a size of 2×2 cm, a comfortable dimension to paint without wasting pigments, and at the same time a size that allows having a high enough number of pixels in the final spectral cube, as the next paragraphs will show.

**Figure 3:**
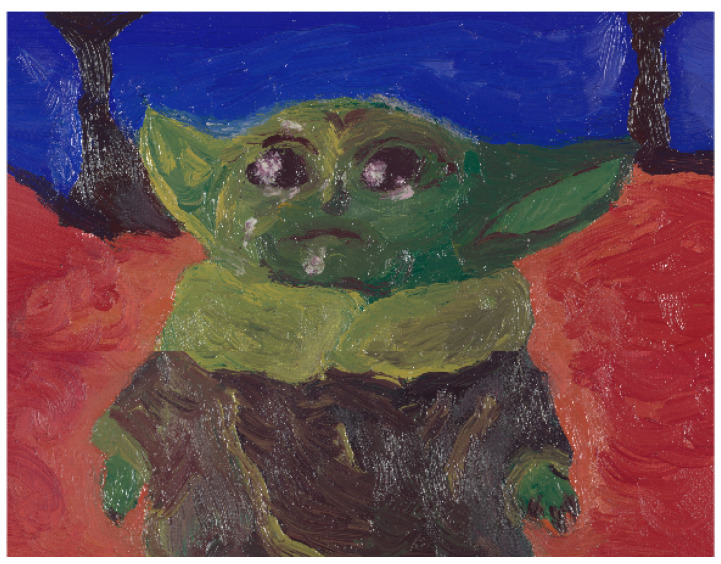
Mockup painting realised with the same set of pigments used for the composition of the mixture samples. Reproduced from [47] with permission from the AIC.

As a validation test, a homemade mockup painting was realised for the occasion, using the same set of pigments (Figure 3).

**Figure 4:**
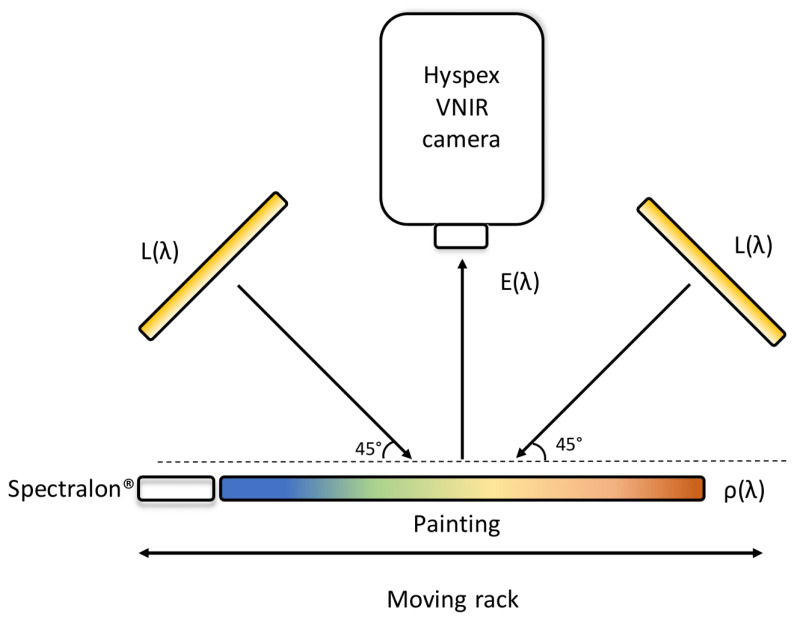
Hyperspectral image acquisition setup. The light sources are placed at 45°, while the camera is at 0°. This allows avoiding specular reflection and shadows. In the push broom setup, the translator stage slides across the field of view of the camera at a speed synchronised with the integration time of the camera.

Hyperspectral images of the objects prepared for this research were captured using a push broom hyperspectral camera HySpex VNIR-1800 produced by Norsko Elektro Optikk. This line scanner uses a diffraction grating and results in generating 186 images across the electromagnetic spectrum, from 400 to 1000 nm, with a spectral sampling of 3.26 nm. The focus of the optics was set to 30 cm, with a 17° field of view and 1800 pixels per line, which allows obtaining a pixel resolution of approximately 50 m. Considering the 2×2 cm size of a mixture patch, this resolution yields approximately 160,000 pixels per sample. The objects were illuminated by a halogen Smart Light 3900e produced by Illumination Technologies, guided on the scene via fibre optics, projecting lights at 45° with respect to the camera (Figure 4). At each acquisition, a Spectralon ^®^ calibration target with a known wavelength-dependent reflectance factor was included in the scene. The target served to estimate the light source spectrum and to compute the reflectance at the pixel level. The HyspexRAD software was deployed to perform radiometric correction [48]. Flat field correction was performed to correct the spatial non-uniformities of the illumination field. Due to noise present at both ends of the spectrum, the first 10 and last 10 spectral bands are omitted from the data, therefore leaving spectra with 166 data points. The reflectance factor of each patch was obtained by averaging over a manually cropped area. Post-processing of hyperspectral images and the analysis presented in the following sections are conducted using MATLAB (The MathWorks Inc., Natick, MA, USA).

### 2.3. Spectral Unmixing Method

To perform SU, the Nelder–Mead optimisation method for non-linear constrained functions [49] is adopted. The array of concentrations $\hat{C}$, subject to NC and SC constraints, is retrieved by optimising an objective function based on the Mean Square Error (MSE) between two spectra:

(3) $$M\;S\;E\left(Y,\hat{Y}\right)=\sum_{j=1}^{N}\frac{{\left(Y_{j}-{\hat{Y}}_{j}\right)}^{2}}{N}$$

in which $\hat{Y}$ is the estimated spectrum obtained combining the spectral library of pure pigments and the estimated concentration vector $\hat{C}$, according to the evaluated imaging model *f*:

(4) $${\hat{Y}}_{\left[N\times 1\right]}=f\left(E_{\left[N\times q\right]},{\hat{C}}_{\left[q\times 1\right]}\right)$$

The optimisation problem to solve is therefore:

(5) $$\begin{array}{c} argmin\left(\frac{\sum_{j=1}^{N}{\left[Y_{j}-f\left(E_{j},{\left(\alpha_{1},\ldots ,\alpha_{q}\right)}^{T}\right)\right]}^{2}}{N}\right)\\ s.t\;\alpha_{i}\geq 0,\forall i\in \left\{1,\ldots ,q\right\}\;and\;\sum_{i=1}^{q}\alpha_{i}=1 \end{array}$$

Minimising MSE means maximising the spectral similarity between the ground truth measurement and its reconstruction. However, spectral accuracy does not lead to a complete evaluation of an imaging model in the context of pigment identification. Indeed, the accuracy from the concentration standpoint is as, if not more, valuable.

We propose to use a concentration error Δα that can be computed as the Euclidean distance between the ground truth concentration vector *C* and its estimation $\hat{C}$. The MSE and Δα can then be combined to yield a score *w* that considers both spectral and concentration accuracies. In Equation (6) both terms are scaled by their maximum possible value: 1 for MSE, and $\sqrt{2}$ for Δα (bearing in mind the compliance with SC and NC).

(6) $$w=M\;S\;E\;\left(Y,\hat{Y}\right)\;+\frac{\Delta \alpha }{\sqrt{2}}$$

As *w* takes on smaller values, the unmixing is considered more successful. In this way, instances reporting low MSE values and high concentration errors can be penalised in favour of instances with slightly higher MSE values but better accuracy in detecting the correct pigments.

### 2.4. Evaluation Protocol

**Figure 5:**
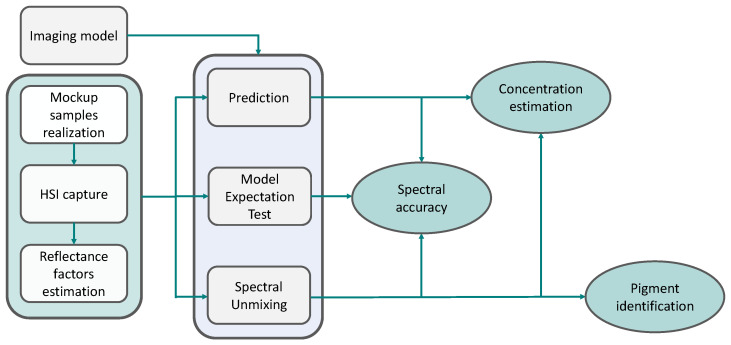
Experimental flow chart. The mockup samples are realised and acquired in a Hyperspectral Imaging (HSI) setup, then the reflectance factors of the patches are estimated (Section 2.2). Each imaging model is evaluated individually. Different features of the imaging models can be observed, depending on the task performed: the model expectation test allows to evaluate the spectral accuracy; the prediction task investigates spectral accuracy and concentration estimation, whereas spectral unmixing comprehends spectral accuracy, concentration estimation, and pigment identification.

The steps taken to evaluate the features of the imaging models are summarised in Figure 5.

Using the spectral measurements and the information contained in the ground truth of the dataset, three tasks were performed to evaluate the properties of the seven imaging models. For all the described tasks, the mixing constant τ of the hybrid models is arbitrarily set to 0.5.

1. **Model expectation test**. For each mixture sample, the models produce spectra using the ground truth concentration vector. This test is used to explore the forward performances of the models and their spectral accuracy.
2. **Prediction task**. In this inversion test, only the information of the pigments included in a mixture is used. All models are inverted in a facilitated unmixing, as the spectral library is pruned down to contain only those pigments. The concentrations are retrieved through the optimisation algorithm, and the spectral reconstruction and the concentration vector are evaluated, via the proposed score *w*. With this task, it is possible to evaluate the ability of each model to retrieve accurate concentrations, given that they are not allowed to select endmembers absent in the mixture, while at the same time keeping a good degree of spectral reconstruction.
3. **Unmixing**. In this instance no prior information is used. All models undergo the task of retrieving the concentrations, starting from the spectrum of the measurement and the spectral library. For each mixture sample, the best model is chosen by selecting the lowest *w* score. With the task of full unmixing, it is possible to evaluate all the characteristics observed in the previous tasks: spectral accuracy and concentration accuracy, plus the ability to detect the correct pigments present in a mixture.

## 3. Results

### 3.1. Model Expectation Test

**Figure 6:**
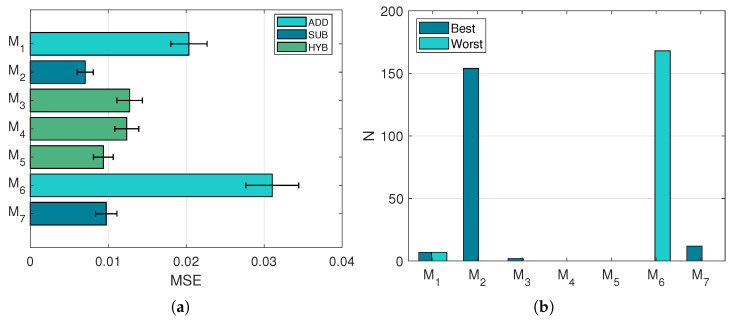
Performances in the model expectation test. (**a**): Average MSE values and respective 95% confidence intervals. (**b**): Number of times each model has been selected as best or worst in terms of MSE.

The spectra output by the investigated models is compared to the ground truth in terms of MSE. An overview of the individual performances of the models is reported in Figure 6. It is observable that there exists a correlation between the MSE values and the nature of the models (Figure 6a): subtractive models fared the best, followed by hybrid models, and then by additive models. Even within the pool of hybrid models, it is clear how the general tendencies of the models affect the ranking, as $M_{5}$ (subtractive-oriented) exhibits better MSE values than $M_{4}$ (additive-oriented). Figure 6b reports the number of times each model was selected as best or worst, depending on the MSE value of the reconstruction.

### 3.2. Prediction

**Figure 7:**
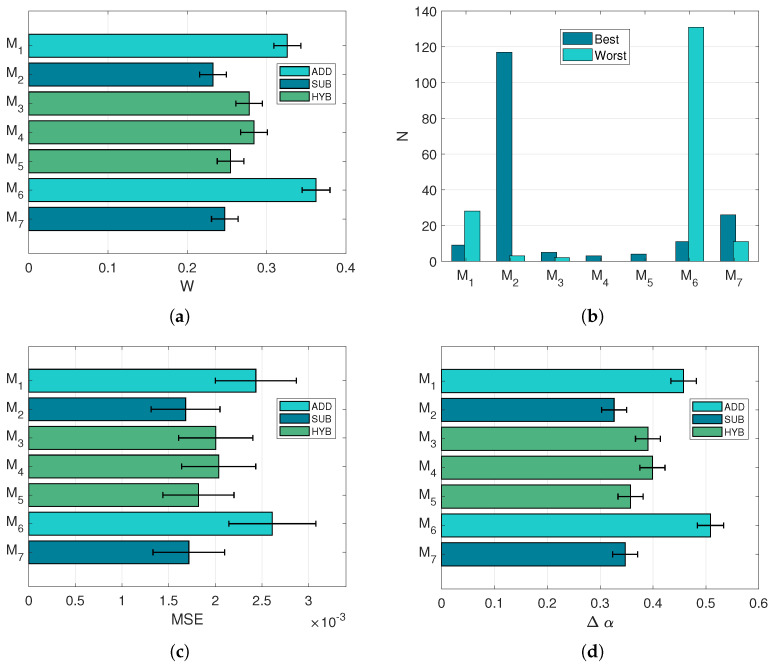
Performances in the prediction task. All error bars refer to 95% confidence intervals. (**a**): Mean *w* score. (**b**): Number of times each model has been selected as best or worst in terms of *w* score. (**c**): Average MSE. (**d**): Mean concentration error Δα.

The task of predicting the concentrations of a mixture, knowing the primaries involved, is performed solving the optimisation problem (Equation (5)). The algorithm is forced to select from either two or three endmembers, depending on the sample mixture analysed. Figure 7 shows correlation between the values of MSE (Figure 7c) and the score *w* (Figure 7d), indicating that good spectral reconstructions yield good concentration retrievals as well. Indeed, the relative differences in the score *w* are amplified, if compared to the individual MSE and Δα differences. The model ranking observed with the model expectation test is confirmed, with the pure subtractive model resulting in the most selected throughout the mixture samples (Figure 7).

### 3.3. Spectral Unmixing

In this task, no prior information regarding the ground truth is exploited, which means that the models can also be evaluated on their ability to identify correctly the pigments present in the mixtures.

**Figure 8:**
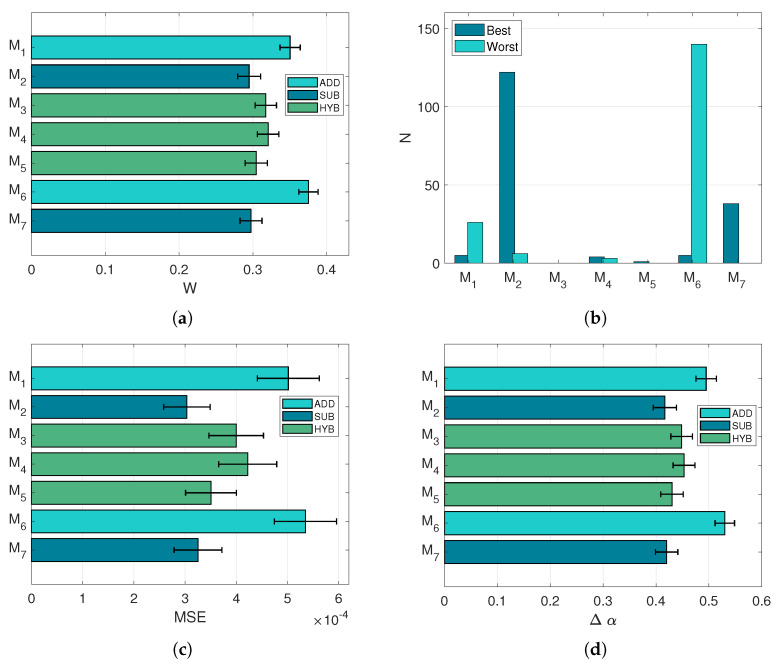
Performances in the spectral unmixing task. All error bars refer to 95% confidence intervals. (**a**): Mean *w* score. (**b**): Number of times each model has been selected as best or worst in terms of *w* score. (**c**): Average MSE. (**d**): Mean concentration error Δα.

The first part of the analysis focuses on the reconstruction of target spectra and the ground truth concentration vectors, following the procedure adopted in the case of the prediction task. In this perspective, Figure 8 exhibits rather similar results to Figure 7: indeed the ranking pattern of the models is exactly the same, with a very similar selection histogram (Figure 8b) as well. However, it is noticeable how the scale of MSE is reduced by approximately a factor 10 (Figure 8c), indicating more accurate spectral reconstructions when the spectral library of endmembers is extended and more pigments can be selected. Incidentally, the concentration error Δα (Figure 8d) is slightly higher than in the prediction task.

Although the MSE values are lower, the reconstructions proposed by the unmixing task cannot be considered better than those of the prediction task, which used solely the pigments contained in the ground truth. The introduction of the score *w* (Figure 8a) is crucial to separate the reconstructions that overfit the spectral data at the expense of the concentration error. Based on these results, the models selected for the study describe partially the mixture of pigments, since very accurate retrievals of concentrations are rarely achieved.

### 3.4. Pigment Identification

An estimated concentration vector rarely presents entries with a value of exactly 0, as spurious concentrations of some endmembers are often output in the optimisation process. It is nonetheless interesting to inspect the detection accuracy of each model: i.e., the ability to identify correctly the pigments present in the mixture ground truth. Knowing that small spurious concentrations can be neglected, we define for each model a concentration threshold $\alpha_{T}$, below which a pigment is considered as not present. In order to do so, the Receiver Operating Characteristic (ROC) curves of each model are analysed. The False Positive Rate (FPR) and True Positive Rate (TPR) are computed varying $\alpha_{T}$ in the interval [0,1] at 0.01 steps. The cut-off value of FPR and its correspondent $\alpha_{T}$ are retrieved using the maximum of the Youden Index *J* [50].

Table 3 reports the cut-off values of $\alpha_{T}$ for each model. Ideally, the lower $\alpha_{T}$ the better, as pigments estimated with a concentration smaller than this value are considered as not detected. In this case, we observe how the hybrid models $M_{3}$, $M_{4}$, and $M_{5}$ perform slightly better than the subtractive ones $M_{2}$ and $M_{7}$. It is worth noting that these values might be too high from a conservation standpoint, as concentrations of around 10% might be significant, but would end up being neglected in this particular instance.

**Figure 9:**
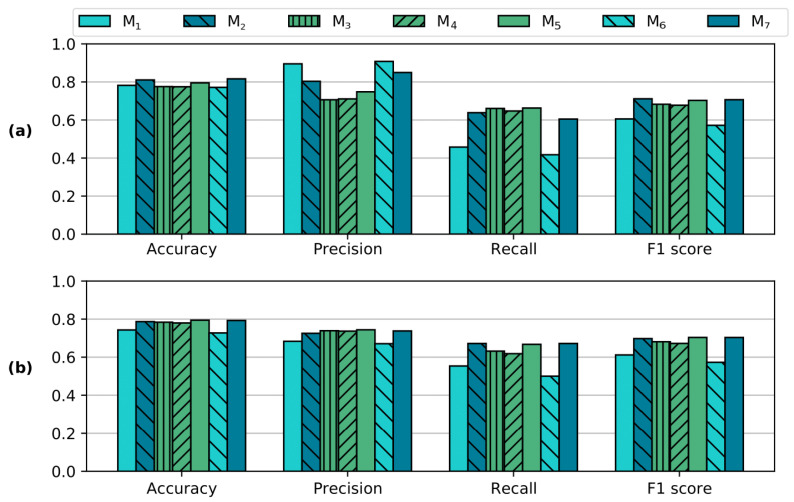
Performances of pigment detection. (**a**): The scores are calculated at the concentration thresholds reported in Table 3. (**b**): The scores are computed at a fixed $\alpha_{T}=0.15$. The overall detection performance is slightly poorer when a fixed $\alpha_{T}$ is used, as it is observable by the small decreases in accuracy. At the same time, the trade-off between precision and recall yields very similar F1 scores in both conditions (**a**,**b**).

The detection ability of the models is analysed via the scores of accuracy, precision, recall, and F1. Figure 9a considers the scores at the concentration thresholds reported in Table 3. The differences in accuracy indicate that the preference should be given to subtractive-based imaging models. Figure 9b exhibits the same scores but this time obtained at a fixed concentration threshold $\alpha_{T}=0.15$, which is selected arbitrarily. In this instance, the scores suggest generally poorer performances than when $\alpha_{T}$ is optimal, while the differences between models are less appreciated.

### 3.5. Mockup Painting

As a validation test, pigment mapping is applied to the mockup painting depicted in Figure 3. To drastically reduce the computation time, the spectral cube was spatially down-sampled by a factor of 10 without performing interpolation, which would have introduced an element of artificial mixing.

**Figure 10:**
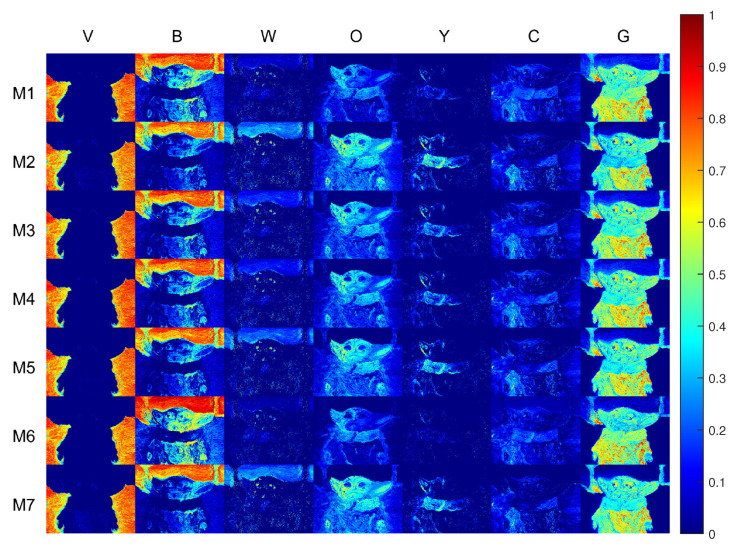
Pseudo-colour concentration maps related to each pigment (by **column**) and investigated imaging models (by **row**). The best performances are obtained by the subtractive-based models, whereas the additive-based models reported the poorest results.

Figure 10 reports the pigment concentration maps retrieved by each model. The imaging models are ordered by rows, while the pigments are in columns.

A few remarks can be made considering the previous knowledge on how the painting was realised. The lower part of the sky contains a significant amount of Kremer White (W) pigment. Such concentration is correctly identified by the subtractive-based models, and not fully appreciated by the additive-based ones. Considering the column of pigment Carmine (C), the additive-based models detect its presence in the right portion of the subject’s face. As a matter of fact, Carmine (C) is not present in this specific area, and its absence is correctly identified by the subtractive-based models. There is a generally strong tendency to misidentify pigment Naples Yellow (Y) with Gold Ochre (O).

From this analysis, the observations made while comparing the imaging models on the mixture samples are confirmed, with the subtractive-based models performing significantly better than their hybrid and additive counterparts.

With the selected unmixing algorithm, the spectral reconstructions showed increasing accuracies as the endmember spectral library was extended. For this reason, a new score *w* was introduced to penalise those instances where the spectral reconstruction is over-prioritised at the expense of the estimation of the pigment abundances. More ways of penalisation can be investigated: the concentration error in its current states does not consider the scale of the concentrations, but only the magnitude of the difference. Moreover, instances where a pigment results to be a false positive could be highly penalised, as misdetections could lead to wrong conservation treatments. However, we also note that a good unmixing does not imply directly a good rendering, as we observed in [47], and future joint analysis of unmixing and rendering are required in order to provide conservation scientists with adapted tools. These problems are also related to the diffusive assumption and future studies should address advanced BRDF descriptions. Indeed, in our mockups we observe the presence of specularities.

## 4. Conclusions

This work proposed the comparison of imaging models in the context of oil painting through an evaluation protocol. This protocol enables the evaluation of the performance ascribable to different properties, namely: spectral accuracy, estimation of the pigment abundances, and pigment identification. Mockup samples of mixtures of pigments in various concentration ratios and a mockup painting were realised for the occasion and acquired in an HSI setup. From the imaging model comparison, we demonstrated that subtractive-based models are to be preferred to their additive counterparts.

A more advanced SU algorithm that exploits the sparsity property of concentration vectors in oil painting could be considered. The imaging models can be improved by augmenting their specificity to oil painting, including more factors such as varnishes, binders, supports, and the impact of the pictorial technique. Also, it would be of interest to apply our protocol to imaging models based on the Kubelka–Munk and the four-flux theories, which characterise light–matter interactions. The directionality of reflection described by BRDFs could also be considered in future work. The expansion of the investigated regions of the electromagnetic spectrum is a direction of future work as well since pigments exhibit renowned properties in the shortwave infrared (SWIR) region.

## Figures and Tables

**Table 1:** Proposed imaging models divided into three main categories: additive (A), subtractive (S), and hybrid (H). The models $M_{4}$ and $M_{5}$ are indeed hybrid but have strong additive and subtractive tendencies, respectively.

Label	Name	Equation	Category
$M_{1}$	Additive	$Y=\sum_{i=1}^{q}\;\rho_{i}\alpha_{i}$	A
$M_{2}$	Subtractive	$Y=\prod_{i=1}^{q}\rho_{i}^{\alpha_{i}}$	S
$M_{3}$	Yule-Nielsen	$Y={\left(\sum_{i=1}^{q}\alpha_{i}\rho_{i}^{\tau }\right)}^{\frac{1}{\tau }}$	H
$M_{4}$	Additive-Subtractive	$Y=\tau \sum_{i=1}^{q}\alpha_{i}\rho_{i}+\left(1-\tau \right)\prod_{i=1}^{q}\rho_{i}^{\alpha_{i}}$	H/A
$M_{5}$	Subtractive-Additive	$Y=\left(\sum_{i=1}^{q}\alpha_{i}\rho_{i}^{\tau }\right)\left(\prod_{i=1}^{q}\rho_{i}^{\alpha_{i}\left(1-\tau \right)}\right)$	H/S
$M_{6}$	LIP additive	$Y=1-\prod_{i=1}^{q}{\left(1-\rho_{i}\right)}^{\alpha_{i}}$	A
$M_{7}$	LIP subtractive	$Y=1-exp\left[-\prod_{i=1}^{q}{\left[-log(1-\rho_{i})\right]}^{\alpha_{i}}\right]$	S

**Table 2:** Pigments included in the set of mockups. The codes refer to the serial number assigned by the manufacturer. The labels identify the pigments and are arbitrarily assigned to better understand the results when the mockup painting is analysed.

Name	Code	Label
Kremer White	46360	W
Ultramarine Blue	45030	B
Naples Yellow	43125	Y
Carmine	23403	C
Vermilion	42000	V
Viridian Green	44250	G
Gold Ochre DD	40214	O

**Table 3:** Concentration thresholds $\alpha_{T}$ computed for each model. The lowest and preferable values are obtained by the hybrid models $M_{3}$, $M_{4}$, and $M_{5}$, indicating that they can discard more confidently false positive detections.

	$M_{1}$	$M_{2}$	$M_{3}$	$M_{4}$	$M_{5}$	$M_{6}$	$M_{7}$
$\alpha_{T}$	0.26	0.18	0.13	0.13	0.15	0.30	0.20

## Data Availability

The data presented in this study are available in the Supplementary Material.

## Supplementary Materials

The following are available at https://www.mdpi.com/article/10.3390/s21072471/s1.

## Abbreviations

The following abbreviations are used in this manuscript:

MSI	Multispectral Imaging
HSI	Hyperspectral Imaging
SU	Spectral Unmixing
SC	Sum-to-one Constraint
NC	Non-negativity Constraint
CH	Cultural Heritage
PM	Pigment Mapping
BRDF	Bidirectional Reflectance Distribution Function
LIP	Logarithmic Image Processing
MSE	Mean Square Error
ROC	Receiver Operating Characteristic
FPR	False Positive Rate
TPR	True Positive Rate
